# Dynamics of non-communicable disease prevention, diagnosis and control in Lebanon, a fragile setting

**DOI:** 10.1186/s13031-020-00337-2

**Published:** 2021-01-11

**Authors:** Nadine Zablith, Karin Diaconu, Farah Naja, Maria El Koussa, Giulia Loffreda, Ibrahim Bou-Orm, Shadi Saleh

**Affiliations:** 1 NIHR Global Health Research Unit on Health in Situations of Fragility, Musselburgh, UK; 2 Global Health Institute, American University of Beirut, Beirut, Lebanon; 3 Institute for Global Health and Development, Queen Margaret University, Edinburgh, UK; 4 Nutrition and Food Sciences Department, Faculty of Agriculture and Food Sciences, American University of Beirut, Beirut, Lebanon

**Keywords:** Fragility, Non-communicable diseases, Prevention, System dynamics

## Abstract

**Background:**

Non-communicable diseases (NCD) present an increasing global health challenge, particularly for settings affected by fragility where access to care may be disrupted, and where high-quality continuous care delivery is difficult to achieve. This study documents the complex dynamics of NCD prevention and management in the fragile setting of rural Beqaa, Lebanon.

**Methods:**

Participatory system dynamics methods were used, including 30 semi-structured interviews and three Group Model Building (GMB) workshops. Participants included health care providers offering NCD care, and Lebanese host- and Syrian refugees community members affected by NCDs.

**Results:**

Participants across all groups articulated a shared complex understanding of both the structural and direct determinants behind NCD onset. Lebanese and Syrian community members further identified several barriers to health seeking, including restrictions in health coverage, limited availability of services in the Beqaa and perceptions of poor-quality care. Health providers and community members described a health system overtly focused on disease control and overwhelmed by delivery of care to people living with NCD across both communities.

**Conclusion:**

Participants across all groups agreed on the need for health promotion and primary prevention activities and identified priority interventions in these areas.

**Supplementary Information:**

The online version contains supplementary material available at 10.1186/s13031-020-00337-2.

## Background

Fragility is a multidimensional phenomenon which constitutes an increasing threat to achieving the Sustainable Development Goals [1]. The threat relates to setting specific characteristics, such as the presence of violence and conflict [2], or the state’s limited institutional capacities, which compromise health systems functions [3]. However, in relation to health in particular, fragility is increasingly used to describe the precarious relationships between the health system and its served communities. When this relationship lacks trust, health seeking and appropriate utilization of services is challenged [4], compromising health outcomes.

Lebanon, an upper middle-income country [5], has frequently been fragile given the country’s extended periods of political upheaval and considerable governance and economic development challenges [6]. Regional instability, marked by the Syrian crisis, has contributed to an influx of refugees to an already growing Lebanese population [7]; of the 6 million people residing in Lebanon, around 1.5 million are Syrian refugees [8]. The rural area of the Beqaa hosts the highest percentage of Syrian refugees (36%) [7] and is considered to be in major need of health institutional support [7]. Prior to the Syrian crisis, the Beqaa was amongst the most deprived settings in Lebanon [9] and, years into the crisis, it is still among the poorest areas in the country [10]. The bulk of the Syrians in Beqaa live in informal tented settlements and suffer from precarious living conditions which may increase levels of stress, poor hygiene, and associated health deterioration [11, 12].

The healthcare system in Lebanon is highly fragmented, with healthcare being primarily provided by the private sector [13]. Six social insurance funds exist to cover health needs under the tutelage of different government bodies [14]. However, approximately half the population has no formal health coverage and is eligible for support from the Ministry of Public Health for hospital care only; for any non-urgent or critical issues care is sought from private clinics or non-governmental organisations (NGO) - supported centres for which out of pocket payments apply [15]. Out-of-pocket household expenditures remain the main contributor to health financing [16]. The Syrian crisis and influx of refugees placed a significant burden on the healthcare system in Lebanon [17]. Health utilization for Syrian refugees is reimbursed by the United Nations Refugee Agency (UNHCR), however refugees access services in the same primary health facilities as the Lebanese population [18]. Hospital care is supported only for obstetric and life-threatening conditions with 25% out of pocket payment of hospitalization costs [7].

In Lebanon, the burden of NCDs – including cardiovascular diseases, diabetes, cancer and chronic respiratory diseases – remains the largest component of the country’s health profile with 91% of all deaths attributed to NCDs [19]. The prevalence of modifiable NCD risk factors (e.g. physical inactivity) has been increasing among the Lebanese population [20–22] and is similarly high among Syrian refugees [23]. Initiatives to address the NCD burden are ongoing. In 2016, the Ministry of Public Health (MOPH) developed a national NCD prevention and control plan (NCD-PCP) [24, 25], but failed to implement it. Currently, the health policy in Lebanon therefore fails to adequately address issues of NCD prevention [26] with policy on NCD care provision also being in its infancy [27].

To achieve the sustainable development goals (SDGs) – including on the prevention and treatment of NCDs by 2030 (SDG target 3.4) [28] – both the delivery and utilization of comprehensive NCD care is necessary. Little is known about how to secure this in fragile settings. This study discusses the dynamics of health seeking, health service utilization and delivery from the perspectives of service users (both host and refugee communities) and health providers in one of the most fragile areas of Lebanon. We offer reflections on health seeking patterns among these populations and identify critical gaps in service delivery which, if addressed, could assist the Lebanese system to curb the rising NCD burden.

## Methods

### Design

Participatory system dynamics methods [29] were used to identify influences on the prevalence of NCDs, and the factors affecting their prevention and management, as perceived by health providers and Lebanese and refugee communities residing in the Beqaa.

The study included semi-structured interviews followed by Group Model Building (GMB) workshops [30]. The GMB sessions included a series of exercises, each based on a specific script (Additional file 1), which were refined based on emerging insights from interviews. Exercises and scripts for community participants focused on eliciting perceptions on: i) the characteristics of persons suffering from chronic conditions, ii) the factors leading to the onset of such conditions and iii) the help- and health-seeking journey of people living with NCD (i.e. documenting the resources that affected persons would approach for assistance in order to cope with or improve their health once they perceive being ill). Health providers additionally reflected on the capacities of the health system to address NCD.

### Participants

Three participant groups in the Beqaa area were targeted for participation in both interviews and workshops:

(1) Health care providers involved in NCD care for both Lebanese and refugee communities,

(2) Adult Lebanese host community members interested in NCD, including people living with, or acting as caregivers for other persons with, NCDs/risk factors, and.

(3) Adult Syrian refugee community members interested in NCDs, including people living with, or acting as caregivers for other persons with, NCDs/risk factors.

Recruitment of participants was conducted along snowball sampling principles, through contacting stakeholders in the Beqaa, principally the administrators of the Primary Health Care Centres (PHCCs) and the “mukhtars” (heads of district offices). Researchers contacted both these latter stakeholders and asked for recommendations on potentially interested health staff and community members. Researchers then directly approached potential participants, and upon obtaining consent for their participation (written in the case of interviews, and oral in the case of GMB workshops), further asked participants to identify other potentially suitable participants. In recruiting community members, we considered participants’ age and gender balance (ensuring participants of different ages and genders were represented), and the representation of different socio-economic strata. In addition to these criteria, for host communities, we further attempted to sample persons with and without coverage via existing health coverage schemes.

### Data collection

Researchers trained in the use of qualitative methods collected data between February and March 2019. Overall, we collected data from 67 participants. In total, 30 semi-structured interviews were conducted: 10 with health care providers (physicians, pharmacists, nurses, PHCC managers, 5 male) offering chronic disease care at PHCCs in the Beqaa, 10 with Lebanese (3 men, age range overall 23–60) and 10 with Syrian refugee (3 men, age range overall 30–60) community members. Interviews were conducted either at the health care center or a location chosen by participants; predominantly, participants preferred to be interviewed at the health center. Overall, all community participants suffered from a chronic condition or self-identified as being at risk of NCD development.

In addition, three one-day GMB sessions were conducted at the American University of Beirut (AUB). In the first GMB, 10 health care providers (one physician, two pharmacists, six nurses, and one PHCC manager) offering chronic disease care participated; overall participants had between 3 and 15 years’ experience of working in the Beqaa. In the second and third GMB 12 Lebanese community members (41% male, age range 20–50) and 15 Syrian refugees (13% male, age range 24–55) participated, respectively. All community participants self-identified as having an NCD or a risk factor. For GMBs with community members, we convened one workshop, but split participants into sub-groups by gender, and in case of the Syrian refugee community group, we also requested one mixed gender group work on comparing the current experiences in Lebanon to those in Syria. Following completion of each activity, groups were encouraged to present to one another, with facilitators clarifying potentially salient gender-driven differences surrounding the topics of study.

All interviews and GMB workshops were conducted in Arabic, by native speakers, with English translation as necessary for English speaking members of the research team during workshops (only provided during summative parts of workshops so as not to disrupt flow).

### Analyses

Analyses sought to identify the dynamics influencing help- and health-seeking, service utilization and delivery, from the perspectives of our distinct participant groups.

Semi-structured interviews were simultaneously transcribed and translated to English by a professional translator. Translation prioritized meaning and transcripts were verified by members of the research group speaking both Arabic and English.

Research team members (ZN and GL) coded all interviews inductively, with KD routinely reviewing coding practice. The analysis team (ZN, GL, KD) then iteratively identified emerging categories and themes, sense-checking these at routine intervals with all authors; ZN speaks both English and Arabic and the majority of authors of the manuscript are native Lebanese citizens. Causal loop diagrams (CLDs) developed in the GMB workshops underwent a process of refinement. Based on field notes and session recordings, and in line with best practice in GMB [31], we reviewed and clarified variable names and meanings, renamed variables to ensure positive meanings were captured, and refined link polarities.

In total, seven CLDs were developed based on the work of the following GMB sub-groups: two Syrian refugee groups discussing their experiences around NCDs in Lebanon; one Syrian refugee group comparing the NCD health seeking and care experience between Lebanon and Syria; two Lebanese host community groups discussing their experiences around NCDs; and two health care providers groups discussing health seeking, but focusing on NCD service delivery and health system dynamics surrounding this. For sessions where two or more CLDs were developed, we critically compared diagrams to each other to determine differences, and proceeded to merge diagrams if no significant differences were apparent. In most cases, differences existed, however the overarching dynamics discussed were similar: we therefore proceeded to merge these sub-models into one CLD and to highlight divergent pathways/variables. After this process of consolidation, 3 CLDs were available: one each for the Lebanese host community, the Syrian refugee community and the health care providers (see Additional file 1 Figs. 4, 5 and 6). All CLDs were transferred into electronic versions using Vensim [32] software.

Further, we abstracted information from the thematic interview analyses, and triangulated these with the consolidated CLDs, in order to identify main feedback loops and leverage points driving the behaviour of the system. Based on this triangulation, we developed a fourth CLD which describes the overarching dynamics of NCD onset, health seeking and health service delivery in the Beqaa. This latter diagram forms the basis of our presentation of findings.

### Ethics

Ethical approvals were secured from the institutional review boards of AUB and Queen Margaret University, Edinburgh. Participants for all GMB workshops were offered reimbursements for the transport to the workshops and additionally compensation for the time spent at workshops.

## Results

We present summative findings organized around three categories reflecting the dynamics of NCD onset, health seeking and utilization, and whole systems for health describing the NCD landscape in the Beqaa, Lebanon.

### Dynamics of chronic disease onset are driven by governance challenges, availability of financial resources and cultural norms

**Fig. 1:**
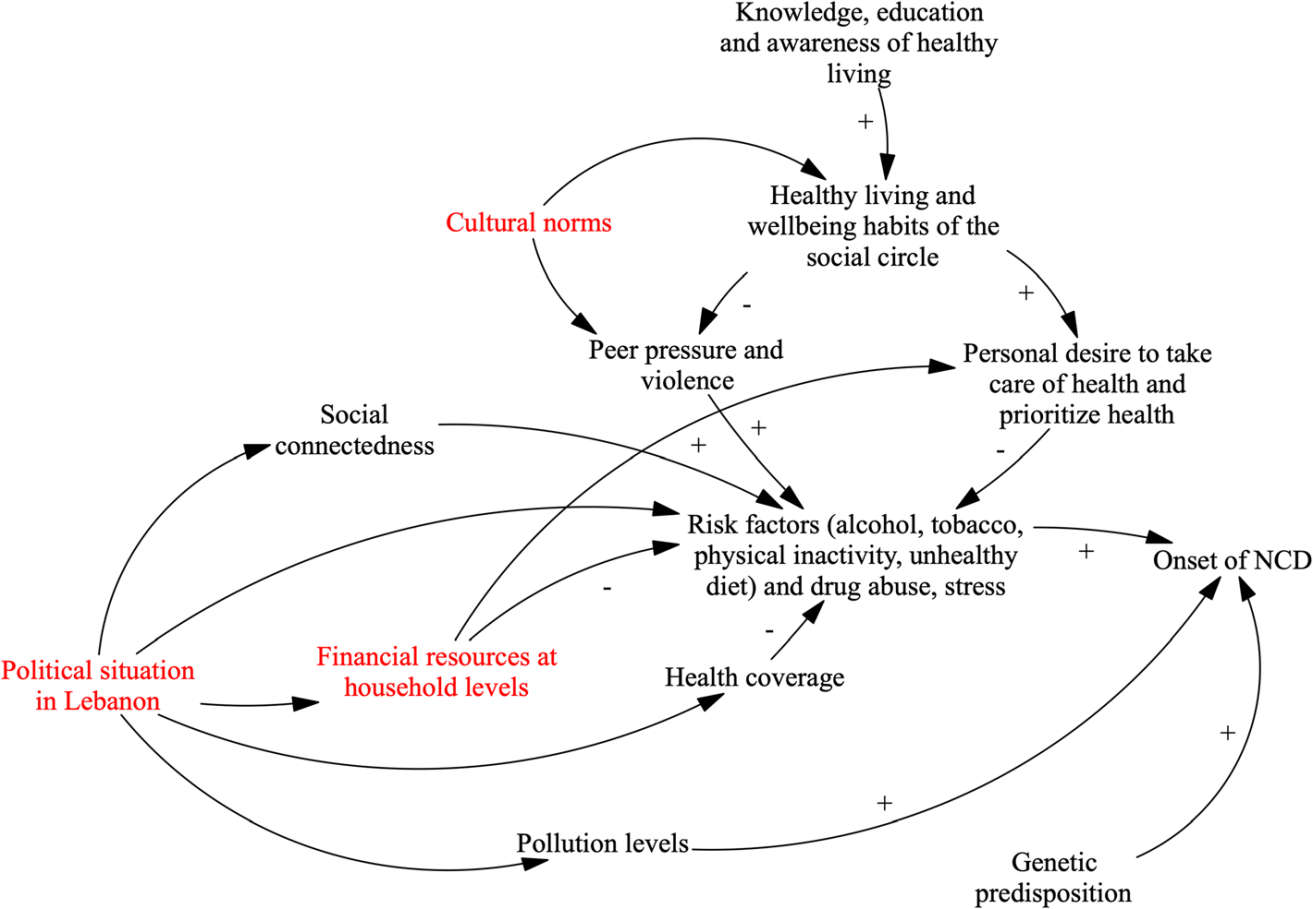
Dynamics of chronic disease onset. Arrows in the figure refer to causal connections between variables, as perceived and described by workshop participants. Where arrows indicate a polarity (+/−), this implies positive or negative associations between variables respectively. For example, the + arrow from “Genetic predisposition” to “Onset of NCD” indicates that participants link the presence of genetic predisposition to increased risk of NCD onset. Where polarities are missing from arrows, participants described that variables related to one another but noted that it was not possible to infer either a positive or negative relationship. Variables in red denote points of fragility as identified by participants

Participant accounts across interviews and workshops focused both on the direct factors affecting NCD risk as well as wider distal and structural elements impacting on the likelihood of persons being exposed to, or developing, risk factors or chronic conditions (see Fig. 1). We note four principal themes emerging across workshops and interviews.

#### Awareness of healthy living is high, however cultural norms and habits of the social circle compromise healthy lifestyles

Participants across all three GMB groups and interview participants showed high levels of awareness of NCD risk factors, naming a variety of lifestyle and genetic factors which contribute to NCD onset (Fig. 1).

*“When I think of an unhealthy lifestyle, I think of fast food, shisha, cigarettes, stress and social conditions”* (Lebanese community member, interview).

*“Minimal physical activity, leads to NCDs … the alcoholic drinks”* (Syrian refugee, GMB).

Participants across community groups in particular noted that their social circle and prevailing cultural norms in the Middle East shaped personal exposure to risk factors. Social activities and encounters in Lebanon feature opulent meals, and smoking of narguileh or cigarettes are firmly embedded traditions.

*“The social activity, the pressure of the friends, the awareness, the traditions. The awareness and traditions affect the smoking and narguileh”* (Lebanese community member, GMB).

The Lebanese host community recognized that social connectedness, and consequently the dissemination of lifestyle advice within ones’ social circle (among peers and from social media) had recently increased persons’ awareness of health living, and the need to seek care and engage in healthy living practices.

*“For instance, some friends tell him, we went through this state before, they tell him such and such. Of course, it is related to being aware as well. If he has high awareness, he will know that this is a symptom”* (Lebanese community member, GMB).

In addition to social media and its effect on the ease to access health information, Syrian refugees recognized that ones’ personal level of education also affected awareness of healthy living. Health care providers agreed that social norms influenced attitudes and practices and also identified media coverage on healthy lifestyles as a potential modifier of unhealthy living.

*“They can go to social media to get information”.* (Lebanese community member, GMB).

Communities reflected on the link between social norms, mental and psychosocial health and healthy living.

*“I: What do you think are some habits that people adopt that could lead to diabetes, heart disease, or hypertension?*

*CM: Sadness, smoking, and shisha. “(Syrian community member, interviews).*

In GMB workshops, community members in particular also spoke about the norms around domestic violence which affect the wellbeing habits of the social network. For instance, when the individual witnessed violent behaviours amongst his family or friends, s/he tended to replicate these behaviours. Peer relationships both inside and outside the home will be affected, leading to the occurrence of peer pressure, domestic violence and bullying. This precedes sadness, potential isolation and stress, ultimately leading to individuals practicing unhealthy lifestyle practices such as smoking, consuming unhealthy foods, using drugs, performing minimal physical activity.

#### National governance affects a family’s ability to practice healthy living

Both Lebanese host community members and health care providers spoke at length about the wider structural determinants behind risk factor emergence, referring specifically to national governance issues which they perceived to lead to societal challenges and, more concretely, to affect financial resources at household and personal levels (see left hand of Fig. 1). For example, participants across all groups recognized that high levels of corruption in the Lebanese government, and further limited employment regulation, impeded economic growth and favored discriminatory employment practices: Syrians are officially not allowed to work, however are informally employed for lower salaries, particularly in the Beqaa.

*“There is no work. People spend their day on the couch and on the internet. Most people hire Syrian instead of Lebanese. Instead of paying a Lebanese 700,000 LBP, they would pay 2 Syrians 300,000 LBP each. So, there are no job opportunities anymore.” (Community member 1 Sadnayel).*

In the long run, this situation has led to the depletion of financial resources at household levels and in turn this further prompted persons to engage in unhelpful coping mechanisms (e.g. tobacco consumption) and restricted the access to a healthy diet and physical exercise (the latter is mainly accessed via payment given limited green and free exercise spaces in Lebanon).

#### Governance challenges directly relate to health coverage and health protection

Health care providers further mentioned that health coverage was directly linked to governance issues. Participants in the provider GMB workshops reported that citizens’ social welfare had been neglected on the policy agenda of Lebanon’s governments over the years, leading to persistent gaps in coverage and increased emphasis on patients paying for care themselves. Health providers predominantly described this as the government not acting in the interest of their population:

*“You are discussing things that need good governance. There is no government, everyone steals from people. I am not going to tell you that the minister has been doing a good job. His work is not good …*. *The system in Lebanon is so corrupt, and if anyone from civil society tries to do something, they fight them”.* (Health care provider, interview).

Absence of health coverage was emphasized as contributing to stress at individual and family levels given the already limited financial resources families have available to pay for care, and consequently exacerbated the financial and mental health burden of persons living with NCDs (see center of Fig. 1). Participants further explained how the absence of comprehensive health coverage schemes also implied that persons did not have access to lifestyle counselling or other health promotion and prevention services. Therefore, persons perceived that the health system was not working towards preventing NCD onset.

Similarly, participants across all groups noted that governance failures also affected the availability of food and environmental policies, and therefore also risks introduced through diets and the (lacking) management of pollution (see bottom of Fig. 1).

*“I would also say it’s about how factories manufacture food and add harmful substances. For example, when manufacturing dairy products, harmful material are added to preserve the food. There is also the habits that people have in their own houses whether personal hygiene or hygiene in general.” (Syrian male).*

#### Structural interventions are a priority for targeting dynamics of disease onset

Across workshops, most participants felt that many aspects of reducing chronic disease development were beyond ones individual control. For example, while participants acknowledged lifestyle changes were possible and within most people’s ability, participants (and particularly health providers) spoke of the need to ensure that the wider social and physical environment is conducive for enabling change – e.g. via healthy lifestyle enabling policies, including tobacco control, environmental regulation and rural planning (e.g. ensuring appropriate waste management and availability of green spaces).

### Dynamics of care seeking and service delivery are shaped by affordability, perceptions of care quality and community trust in services

**Fig. 2:**
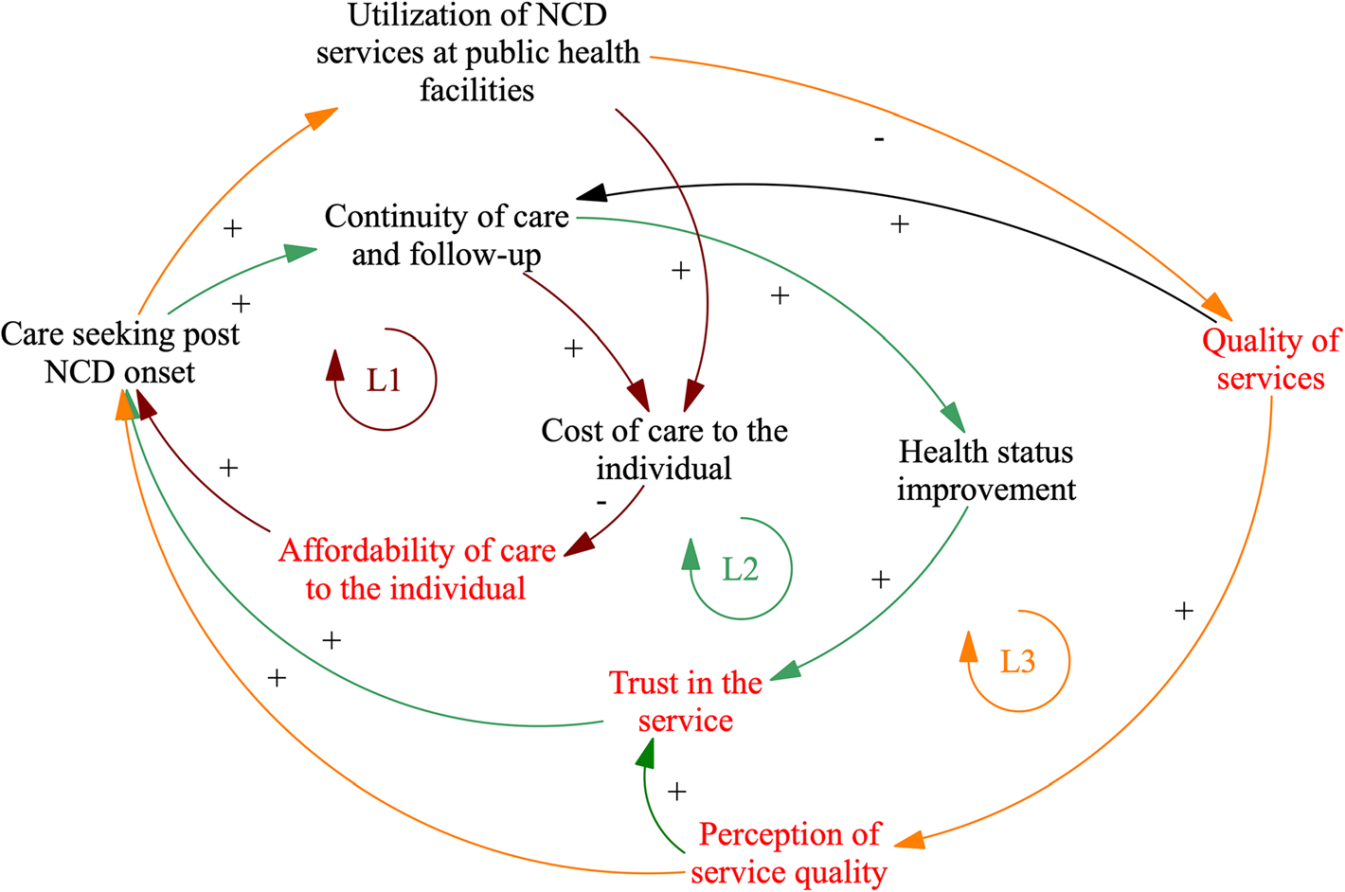
Dynamics of care seeking and service delivery. Arrows in the figure refer to causal connections between variables, as perceived and described by workshop participants. Where arrows indicate a polarity (+/−), this implies positive or negative associations between variables respectively. For example, the + arrow from “Quality of services” to “Continuity of care and follow-up” indicates that participants link higher quality of care to higher likelihood that a patient would be followed up and receive continuous care for their NCD. Where polarities are missing from arrows, participants described that variables related to one another but noted that it was not possible to infer either a positive or negative relationship. Colour is used in the above diagram to indicate the presence of feedback loops. Three loops - depicted as L1-3 (dark red – Loop 1, green – Loop 2, orange – Loop 3) are visible in the diagram and are further explained in the body of the paper. Variables in red denote points of fragility as identified by participants

We distinguish three main feedback loops that characterize the dynamics of care seeking and service delivery (see Fig. 2). Across GMB workshops and interviews, participants reflected that unlike for acute care seeking, NCDs required them to make both an initial decision to utilize health services (usually once an NCD had resulted in serious impairment to day to day life) and then a further continuous decision to utilize services (see Care seeking and Utilization variables in Fig. 2).

#### Loop 1 (Fig. 2, dark red): care seeking compromised by prohibitive care costs

All participants described severe financial constraints at household levels given the limited availability of employment and economic growth in the Beqaa in particular. Participants also spoke at length about the affordability of health care services. In particular, persons noted that for NCDs repeat utilization of health services is necessary: in the absence of health coverage schemes repeat utilization raises the cost of care to the individual in the long term and this compromises affordability. See Fig. 2, pathway of red arrows, noting that continuity of care leads to increases in cost of care to the individual which in turn compromises the overarching affordability of care for individuals.

*“Every doctor you might go to asks for a 50 USD consultation fee. Not everyone can afford that. The ministry of health does not cover everything, and some hospitals do not admit you unless you have insurance. Some people wait until they get paid by the beginning of the month.”* (Lebanese community member, interview).

Vulnerable Lebanese persons that are not covered by the existing health insurance funds (e.g. National Social Security Fund, Civil Servants’ Cooperative or Military Health Funds) are unable to afford outpatient care at private clinics and may instead access pharmacies or civil society run health centres. However, even in discussions about these, the mutual understanding among participants was that the cost of care for NCDs to the individual is high, and that this compromised affordability of care and implied persons dropping out of care. A minority of participants also reported paying for alternative support sources such as traditional medicine or religious persons (data not shown); in these cases, a delay in seeking formal healthcare was noted, with persons acknowledging that seeking care late implied the condition would exacerbate over time.

*“I: What other factors might delay someone in actually going to the healthcare system?*

*CM: The financial situation is one reason. There is no insurance, social security, or ministry coverage. People know they would not be admitted. At the end of the day, no one worries about your health more than you do, but you might not go if you know that you won’t be admitted. This is when people decide to treat themselves. They start having herbal tea and herbal medicine. They think this is how they will get better, and unless there is an emergency, people don’t go to the hospital. As for services like hospitals and healthcare centers, they vary between here and Central Bekaa. The services are good when you have money.” (Lebanese community member).*

Syrian refugees noted gaps in coverage specifically for chronic conditions such as cancer care. Refugees attributed this to humanitarian assistance gaps and noted that they often delay seeking care in order to manage scarce resources; this frequently results in persons presenting at secondary care with complications, incurring higher out of pocket expenditures which potentially lead to catastrophic expenditures. Further, Syrian refugees spoke about potentially choosing to return to Syria to seek care, or of planning to emigrate to European countries.

*“Some people really cannot afford medication. They are begging and going to people’s houses to ask for money. This is actually happening. They tell you that they have a chronic disease.”* (Lebanese community member, interview).

*“Here? In Lebanon? It’s all about money, if you pay, you get your services … .. It’s all about finances.”* (Syrian refugee, interview).

In discussion around help- and health-seeking behaviours, both Syrian refugees and Lebanese community members noted that while seeking care from doctors may be prohibitive in Lebanon, care-seeking from pharmacists was generally affordable and also trusted. Most GMB participants noted that upon developing NCD symptoms, their first point of interaction with the health care system was generally the pharmacy. Pharmacists were noted to assess the case of each person and provide medication. It is only once this avenue is exhausted, and if the patient does not perceive any improvement, that he/she may then seek alternative care.

*“We feel the symptoms, we go to the pharmacy. But the individual feels the symptoms, he feels scared and anxious, then he goes to the pharmacy”* (Syrian refugee, GMB).

*“They usually go to the pharmacy first … It’s because they would save on doctor’s fees. So, they go straight to the pharmacist and get diagnosed and tested there”* (Lebanese community member, interview).

#### Loop 2 (Fig. 2, in green): trust is a critical mediator of care-seeking

In addition to cost of care, trust in the health service – as influenced by patients’ perceptions of care quality or having experienced or heard of health improvements from other persons who had accessed care– plays an important role in whether care is sought again. In Fig. 2, see green arrow pathway, highlighting how health status improvement or perceptions of care quality influence the overarching trust in the health system and therefore continued service utilization.

However, participants across all workshops perceived that improvements in an individual’s health status were unlikely to occur because continuity of care is currently compromised. Barriers which compromise trust in the health system include: the cost of care to the patients, the limited availability of facilities in the region, limited availability or ability to secure medication at facilities, limited availability of equipment to help with accurate diagnosis and routine testing at facility levels. Participants noted that some of these issues are due to current health system governance because resources were likelier to be concentrated in Beirut where access to specialized care was also available.

*“Well, most people who are of the rich class here go to Beirut because they trust the big hospitals like [ …*.] *…*. I *don’t think it is a false perception because a university or research hospital is different from a regular one. Doctors who graduate and work in the Beqaa don’t update their information much.” (Health care provider, interview).*

*“I don’t trust other practitioners. As I told you, they are always in a rush and patients don’t accept this”* (Health care provider, interview).

*“Here in the Bekaa, not everyone trusts the hospitals, so they go to Beirut directly”* (Lebanese community member, interview).

#### Loop 3 (Fig. 2 in orange): quality of care and perceptions of care quality influence repeat care seeking

**Fig. 3:**
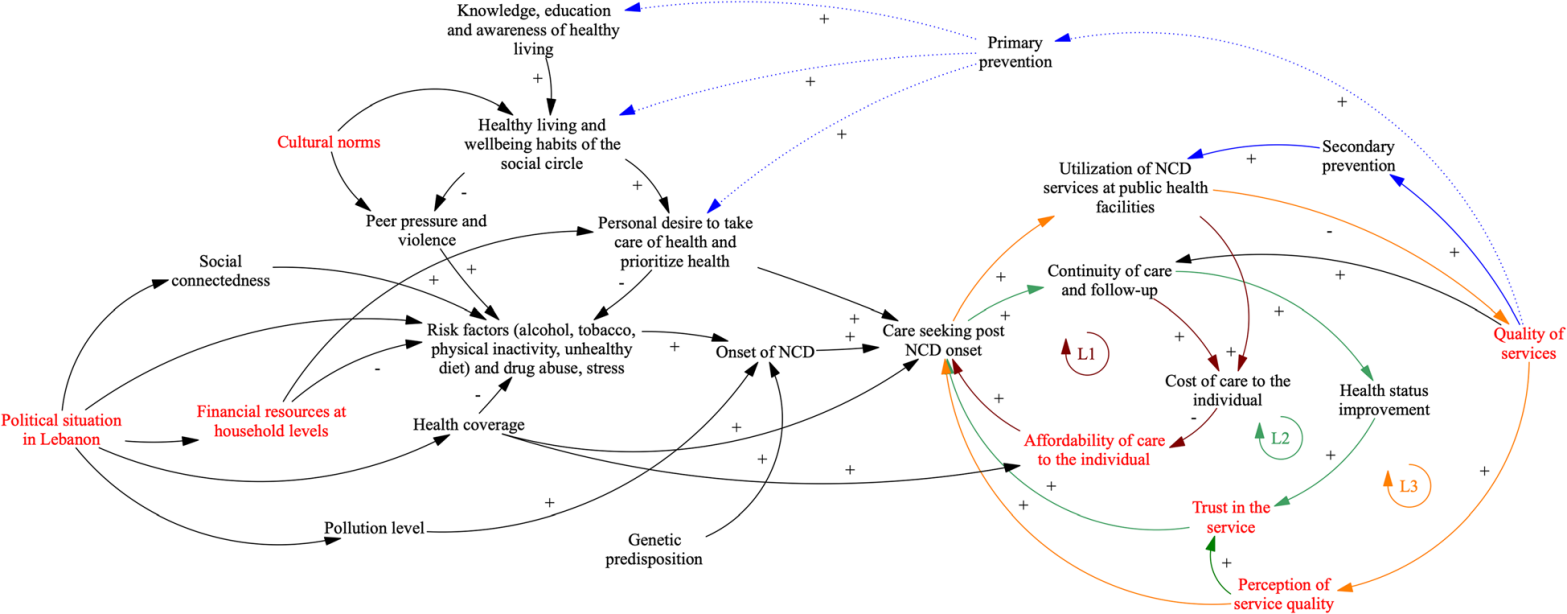
Whole system diagram: Arrows in the figure refer to causal connections between variables, as perceived and described by workshop participants. Where arrows indicate a polarity (+/−), this implies positive or negative associations between variables respectively. For example, the + arrow from “Quality of services” to “Primary prevention” indicates that participants link higher quality of care to emphasis on primary prevention activities within the health system. Where polarities are missing from arrows, participants described that variables related to one another but noted that it was not possible to infer either a positive or negative relationship. Colour is used in the above diagram to indicate the presence of feedback loops. Three loops (dark red – Loop 1, green – Loop 2, orange – Loop 3) are visible in the diagram and are further explained in the body of the paper. Variables in red denote points of fragility as identified by participants. Pathways in blue refer to systemic responses as fronted by the health system: we denote the current prevailing response in a solid blue line (focused on secondary prevention). The dotted line supporting pathways from primary prevention to the distal factors impacting upon NCD onset denotes that participants identified primary prevention as necessary but not implemented widely within the Lebanese health system

The three participant groups noted that both the availability and quality of public healthcare services offered in the Beqaa were poor and that this affected persons’ decision on whether to seek care again (see Fig. 3, orange arrow pathway). In relation to quality of care, participants principally focused on discussing process related indicators such as health facility utilization rates and patient load, consultation times and friendliness of provider communication. Few participants, principally Syrian community members, reflected on quality of care in relation to health outcomes such as NCD control status or prevention of complications; participants within this group noted that the quality of care was better in Syria, where the health system focused more comprehensively on delivery of primary care services, and did so by considering cost-related barriers to care specifically.

*“The healthcare system in Syria was different from the healthcare system here. We cannot ignore that in Lebanon it used to be better; however, there are some key issues you cannot ignore. In Syria, health coverage whether in the private or public clinics, centers, or hospitals was cheap. It was accessible. Doctors, centers, and pharmacies had specific rates irrespective of the disease. So people were not scared of following up on their health. However, here the prices are terrifying for Syrian people.” (Syrian community member).*

Syrian participants also reflected that high utilization among rural clinics placed additional pressure on the health system, compromising quality of care in the long run if no further investments in availability of skilled staff were made (see Fig. 3). Health providers offered similar accounts, emphasizing the limited availability of skilled staff and additionally noting that the Beqaa would experience occasional stock-outs of medication, thus compromising continuity of care.

Lebanese community members further reflected that quality of care differed by facility and by one’s ability to pay for better service, suggesting that care had taken on characteristics of a commercial rather than public good.

*“It depends. In some hospitals, they treat you well. In other places, they treat you like you are nothing. … In some hospitals, treatment is good, but in others it is horrible. It depends on how much you pay them”* (Lebanese community member, interview).

### Whole system model of NCD onset and care delivery in the Beqaa

In GMB discussions, participants were additionally prompted to reflect on intervention priorities surrounding the fragility points identified in their causal loop diagrams (see variables in red in Figs. 1 and 2). Based on a synthesis of insights from these discussions, and recommendations presented by interview participants, we present a whole system model of NCD onset and care delivery in the Beqaa in Fig. 3. Within this model two additions are visible, specifically pathways in blue which reflect systemic responses to the rising NCD burden, focused around primary and secondary prevention activities; we identify two main themes in relation to these pathways.

#### System focused on, and overwhelmed by, secondary NCD prevention

Across GMB workshops, we note that health care provider and community accounts focus on the relationship between current opportunistic screening and detection of cases as part of secondary prevention activities (solid lines in blue, Fig. 3). Providers and community members spoke at length of how persons showing symptoms of NCDs access care (albeit late) and ultimately receive a diagnosis (core of diagram); if patients were then able to afford care, they may be able to control their disease status and maintain their health (the ideal behaviour described by loops 1–3 in Fig. 3). In the presence of health coverage, feedback loop 1 would be balanced and the behaviours of loops 2–4 would imply a system committed to achieving NCD control at individual level.

However, as described in previous sections, the latter situation is very unlikely to occur in practice given limited health coverage and compromised affordability of care, trust and also quality and availability of services in the Beqaa: all of the latter are variables in red in Fig. 3 and have been identified by participants as points of fragility. Health providers in particular reflected on structural elements of care quality which are compromised by high utilization levels (including availability of sufficient medications at health centres). Given such conditions, patients usually fall out of care and health providers are focused on following up on patients as best they can in order to prevent further complications.

*“Primary healthcare centers need actual support from the ministry of health. Support is restricted to a small quantity of medication and supply which is a lot lower than the number of people we get at the center.* “*(Nurse).*

In the long run, especially given the increased utilization of services due to the influx of Syrian refugees, this implies a system that seeks to address the needs of more NCD cases, but at the same time, sees its function and quality compromised due to higher levels of utilization. The absence of health coverage acts as a mitigating factor for demand among Lebanese community members currently; however, should coverage be expanded, health care centres would need to be able to accommodate further patients despite the limited availability of health facilities and human resources in the Beqaa. Given the high influx of Syrian refugees, it is also pertinent to note that the latter may be prioritized for care at public health centres given stable and predictable health financing covered by UNHCR, in contrast to relatively limited health coverage for Lebanese host communities by the state.

*“Healthcare centers are too crowded [ …] I wait too long before getting a turn [ …*]. *As a Lebanese, when you go to the healthcare center, you find that everyone is Syrian. You wait for hours, then you give up and leave … yes … because the practitioners prioritize the Syrians covered by the UNHCR … even hospitals do that, you have to have health coverage”* (Lebanese community member, interview).

While Syrian refugees do benefit from increased generic coverage for NCD services, our participants noted however that funding gaps for specialized treatments in particular still compromise refugee health.

*“For example, if a Syrian refugee needed an operation today other than delivery, they don’t get any support. If someone needs a gallbladder removal, he would need to do it at his own expense. This creates an issue because instead of paying 250,000 LBP or 300,000 LBP, he would need to pay 1,000,000 LBP. For cancer cases, they send the patient all the way to Tripoli. The patient spends a fortune getting there, and the hospital sometimes treats him and sometimes doesn’t.” (Health manager).*

#### Systemic intervention implies a long-term focus on reducing affordability related barriers and on primary prevention

Limited care coverage and an emphasis on secondary detection and prevention alone are unlikely to be sustainable and to be able to address the current issues of high affordability related barriers and limited service quality in the Beqaa. In discussions on how the systems of care around NCDs could be improved, participants across all groups spoke of two main avenues.

First, to support those persons already suffering from NCDs and in order to tackle affordability of care, health coverage should be expanded and especially medication related costs should be covered more comprehensively.

*“I would say that the government and healthcare coverage schemes play an important role. The law set by the government and healthcare coverage instances should be respected by everyone. They all play a role whether it is medical insurance, military coverage, the health coverage for internal security forces, or the cooperatives. (…).*

*I think that the medication for chronic disease should be consistently provided. It is not enough to provide medication 1 out of 10 times. Quality medication for chronic disease should be properly provided” (Health facility manager).*

Second, across all GMB workshops, participants emphasized the need for primary prevention activities (see upper side of Figure dotted lines). Indeed, when mapped out in Fig. 3, it is evident that primary prevention activities could act on the distal drivers behind NCD onset and curb the rising demand for NCD services. This would help with long-term stabilization of health system function by giving the system space to address issues of service availability and quality in the run-up to rising demand. Participants noted that they view primary prevention as an urgent multi-sectoral issue.

## Discussion

Our research has illustrated the potential for using participatory methods, and system dynamics in particular, for studying the complex behaviours at community and health system levels around NCDs. The approach is of particular value when attempting to achieve an understanding of the wider influences on complex phenomena as perceived by diverse groups, ranging from distal factors impacting on NCD onset to health system responses to tackling the rising NCD burden. Our findings indicate the critical missed opportunities for addressing the rising NCD burden in Lebanon and point to the need for expanded health coverage, primary multi-sectoral prevention and health promotion activities. We additionally document the repercussions of restricted health coverage on NCD care seeking, and challenges arising in the case of rapid expanded health coverage for Lebanese communities in particular.

In this study, the GMB discussions and semi-structured interviews provided an opportunity to triangulate findings. Detailed findings on the dynamics of disease onset and care seeking and service delivery Figs. 1 and 2 are available in comprehensive models independently developed by Lebanese and by Syrians community members and by health care providers based in the Beqaa region (data available from authors upon request). Overall, the three models show strong agreement, however, some differences need to be highlighted. For instance, the Lebanese community members and health providers focused on the interconnectedness of the political instability in Lebanon with disease onset and related care seeking and service delivery behaviours.

Our findings indicate that limited financial resources at the household level, combined with inadequate health coverage, influence disease prevention and control, and access and utilization of health care services, and the control of disease. These findings are consistent with the results of previous studies conducted in middle-income countries showing that NCD-related health expenses are devastating and impoverishing [33–35]; further, such findings accord with those specific to health systems attempting to cater to Syrian refugees in particular [36]. In line with our findings, corruption and wider governance challenges have been consistently noted barriers to achieving healthcare access [37], and healthcare quality [38]. A local political economy analysis of NCDs suggests Lebanon’s poor governance features hinder the passing and implementation of health system reforms which could guarantee better accessibility and affordability to NCD services, and strengthen quality of care [39].

Our results further highlighted the complex and dynamic association between the socio-cultural life of communities and NCD risk exposure. Similarly, other studies demonstrated that social networks, traditions and customs affect healthy lifestyle practices, especially in relation to diet [40, 41] and smoking [41–43].

Our results further show that health-seeking behaviours for both Lebanese host community and Syrian refugees are diverse and shaped by a myriad of factors, including perceptions of care quality. These results are in agreement with those obtained in other settings, including via comparable research in Sierra Leone [44]. Participants from both Lebanese and Syrian communities further highlighted that pharmacists are both easily accessible and trusted. This finding aligns with research conducted in Jordan, where Syrian refugees access pharmacies frequently as out of pocket payments were among the lowest in pharmacies [45]. Research suggests that pharmacists can assist in curbing the NCD epidemic if trained appropriate in counselling on disease prevention and NCD related medication prescription [46]. Our research has not focused extensively on the role of pharmacists, and relatively little is known about them; their roles within the complex health system of Lebanon is therefore still to be defined.

This is one of the first studies conducted in Lebanon, using the system dynamics approach for identifying factors affecting NCD onset. System dynamics is particularly suited to the study of complex health issues and systems and has been previously used to research matters such as health care capacity and delivery [47] and health system resilience [48]. In regards to questions specifically associated with NCDs, this study complements recent system dynamics analyses reported in the United States [49, 50], Singapore [51] and Sierra Leone [52].

We acknowledge several limitations. While we have attempted to speak to a diverse set of health providers and community members in the Beqaa, our sampling approach was dependent on snowball sampling and as such selection bias is likely. As with most in-depth qualitative work focused on one region, our work is not generalizable all of Lebanon; however, may be of relevance to areas where large refugee populations have settled and/or that are particularly rural. We have conducted a similar study in the Greater Beirut area and will contrast findings in due course [38]. Merger of participant models and ancillary interpretation of findings is heavily influenced by researchers’ perspectives; however, we have mitigated this by triangulating data with that obtained from qualitative interviews and by openly consulting with diverse team members during write-up.

## Conclusions

A system dynamics approach was used for the first time in Lebanon to identify factors influencing the NCDs prevention, diagnosis and control from the perspective of health care providers, Lebanese community members and Syrian refugees residing in the Beqaa area of Lebanon. This study has identified perceived factors and explanatory pathways related to the dynamics of disease onset, care seeking and service delivery. The study has also highlighted the need for multi-sectoral action in relation to NCD prevention and health promotion. Future research should focus on characterising service quality and coverage among vulnerable populations in Lebanon.

## Supplementary Information

**Additional file 1.** Scripts for group model building workshop.

## Data Availability

The datasets used and/or analysed during the current study are available from the corresponding author on reasonable request.

## Abbreviations

CLDs: Causal Loop Diagrams
GMB: Group Model Building
MOPH: Ministry of Public Health
NCD-PCP: NCD Prevention and Control Plan
NCD: Non-Communicable Diseases
NGO: Non-Governmental Organisations
PHCCs: Primary Health Care Centres
SDGs: Sustainable development goals
UNHCR: United Nations Refugee Agency
