# Supplementary material for: Dynamics of non-communicable disease prevention, diagnosis and control in Lebanon, a fragile setting
Source: Confl Health. 2021 Jan 11;15:4. doi: 10.1186/s13031-020-00337-2 (PMC7802297; doi:10.1186/s13031-020-00337-2)
Supplement: Supplementary file 1 — Additional file 1. Scripts for group model building workshop. [file 13031_2020_337_MOESM1_ESM.docx]

**Appendix 1** **Scripts for Group Model Building Workshops**

| Script 1: GMB with community members | |
| --- | --- |
| Time | Activity |
| 5-7’ | Welcome note (FN)  Scope of NIHR-RUHF project as a collaboration between AUB and QMU  Introduction of team members (QMU and AUB)  Introduction of participants (First name and background)  Introduction to activities:   - Non-communicable diseases: (Cardiovascular diseases, Diabetes, Chronic Respiratory Diseases, Cancer) - Purpose of today: understand more about the populations you serve (both Lebanese and Syrian) and about your experiences in offering NCD care |
| Section I – Activities for variable elicitation (1h15) | |
| 5’  5’  5’  15’ | Rich pictures  Split participants into groups.  Introduce the exercise: we will first focus on people at risk of developing NCDs and then on people affected by these conditions.  Let’s focus on persons at risk and their lifestyle.  Picture 1 – Unhealthy lifestyle  What does “unhealthy lifestyle” mean?  What causes unhealthy lifestyles in the population?  Where are health providers in the picture? (Is there screening, counselling, etc)  Picture 2 – A person with unhealthy lifestyle  Do they seek support for their lifestyle (e.g. to address their risk factors)? (prompt the discussion around 2 options: Yes/No)  Where does the person go to seek support? Why there? What determines where they go?  Are persons able to address their risk factors? (e.g. the patient stops smoking and loses weight) Why? What helps/hinders?  Now, let’s focus on a person with NCDs.  Picture 3 - the journey of patient living with NCD  Draw a person with a typical NCD.  Help-seeking:   - Does this person seek help? Where, when and from whom? What affects whether they seek help? - What happens at the place they seek help? (e.g. diagnosis, medication prescription, counselling etc.)   Health status  Does help result in any improvements? Are persons followed up?  What happens to those living with complications or advanced stages (secondary and tertiary care)?  Group presentations (5 mins on each drawing) |
| 20’ | Reference modes (NZ, AN and R)  Participants will be asked to draw graphs representing the last 10 years. Graphs ought to focus on:  Graphs:   - % of people knowledgeable of healthy or unhealthy lifestyles - Prevalence of NCDs; - % of persons that have chronic conditions who are not maintaining their health (i.e. who are getting worse, have complications, who aren’t controlled)   N.B. These are perceptions not accurate epidemiological figures! Please specify that “prevalence” means ALL people living with the disease, not only diagnosed cases.  Group presentation  At the end, ask them to present the graphs to the audience.  Participants might be invited to think of potential changes around the years 2012-2013 when the influx of Syrian Refugees was very high. |
| Break | |
|  | Variable elicitation  Ask participants to split into the same groups used in the “rich pictures” activity.  Ask each group to use sticky notes and brainstorm top 5 variables for each **(STEP BY STEP)**:  - unhealthy lifestyle  - reasons behind unhealthy lifestyle  - onset of disease and help seeking  - maintaining health  Prompt around: health insurance schemes, wider policy, community and family dynamics, quality of care  N.B. Ask participants to provide definitions for variables if needed. Ensure neutral wording of the variables.  Variables will be compared among each other to ensure **clarity of their meanings** and avoid duplication. |
| 60’ | Give an example on causal linkage of variables  Start with a healthy state and illustrate how people then become at risk:  I.e. first instinct is:  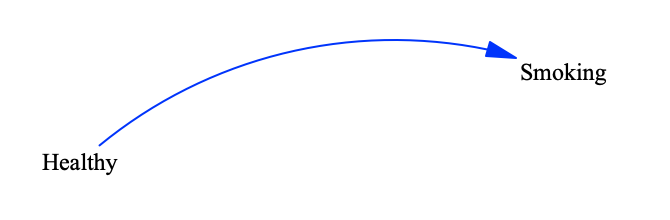  But actually we need to go into a bit more depth:    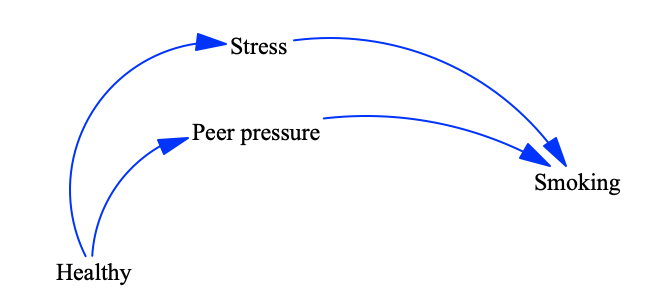  The research team will ensure that the causal links, proposed by the groups, are clear and justifiable by them.  Peer-review of the models by other group members will be encouraged. |
| 20’  30’ | Model presentation and Identification of points of fragility  Each group will present the model and the other groups will be invited to comment on the model.  The research team will ask the participants to identify points of ‘fragility’ in the system and to vote on their severity: 5 votes can be split however they want. |
| 45’ | Lunch |
| 60’ | Identification / prioritization of interventions  We want to know participants’ opinions on where and how to intervene in the models they have just created.   1. What are the elements of a good strategy for intervening? 2. Look at the points of fragility. How would you intervene? 3. Which is the most important intervention?   Giving out stipends for the day  Final payments |

| Script 2: GMB with health providers | |
| --- | --- |
| Time | Activity |
| 5-7’ | Welcome note (FN)  Scope of NIHR-RUHF project as a collaboration between AUB and QMU  Introduction of team members (QMU and AUB)  Introduction of participants (First name and background)  Introduction to activities:   - Non-communicable diseases: (Cardiovascular diseases, Diabetes, Chronic Respiratory Diseases, Cancer) - Purpose of today: understand more about the populations you serve (both Lebanese and Syrian) and about your experiences in offering NCD care   Demonstrating use of GMB methods - KD |
| Section I – Activities for variable elicitation (1h15) | |
| 5’  5’  5’  15’ | Rich pictures  Split participants into groups.  Introduce the exercise: we will first focus on people at risk of developing NCDs and then on people affected by these conditions.  Let’s focus on persons at risk and their lifestyle.  Picture 1 – Unhealthy lifestyle  What does “unhealthy lifestyle” mean?  What causes unhealthy lifestyles in the population?  Where are health providers in the picture? (Is there screening, counselling, etc)  Picture 2 – A person with unhealthy lifestyle  Do they seek support for their lifestyle (e.g. to address their risk factors)? (prompt the discussion around 2 options: Yes/No)  Where does the person go to seek support? Why there? What determines where they go?  Are persons able to address their risk factors? (e.g. the patient stops smoking and loses weight) Why? What helps/hinders?  Now, let’s focus on a person with NCDs.  Picture 3 - the journey of patient living with NCD  Draw a person with a typical NCD.  Help-seeking:   - Does this person seek help? Where, when and from whom? What affects whether they seek help? - What happens at the place they seek help? (e.g. diagnosis, medication prescription, counselling etc.)   Health status  Does help result in any improvements? Are persons followed up?  What happens to those living with complications or advanced stages (secondary and tertiary care)?  Group presentations (5 mins on each drawing) |
| 20’ | Reference modes (NZ, AN and R)  Participants will be asked to draw graphs representing the last 10 years. Graphs ought to focus on:  Graph 1:   - Prevalence of risk factors; - % of people knowledgeable of the harmful effects; - % of people seeking health advice; % of “success stories”.   Graph 2:   - Prevalence of NCDs; - % of diagnosed cases of the total prevalent cases; - % of people living with complications   N.B. These are perceptions not accurate epidemiological figures! Please specify that “prevalence” means ALL people living with the disease, not only diagnosed cases.  Group presentation  At the end, ask them to present the graphs to the audience.  Participants might be invited to think of potential changes around the years 2012-2013 when the influx of Syrian Refugees was very high. |
| Break | |
| 5’  5’  5’  5’ | Variable elicitation  Ask participants to split into the same groups used in the “rich pictures” activity.  Ask each group to use sticky notes and brainstorm **(STEP BY STEP)**:  - factors affecting the occurrence/prevalence of NCD **[Pick top 5]**  - factors affecting providers’ abilities to keep people adopting a healthy lifestyle and seeking routine health check-ups and preventative services; **[Pick top 5]**  - factors affecting NCD risk reduction practices (including smoking; diet; physical activity, overweight/obesity and alcohol use); **[Pick top 5]**  - factors affecting disease diagnosis and control. (15 minutes) **[Pick top 5]**  If needed, prompt participants to identify variables regarding the current knowledge status of health providers.  Encourage them to brainstorm: how could these patients’ experiences change according to health coverage schemes (MOPH, Social Security Fund, Private Insurance, etc) and/or nationality (Lebanese vs refugee)? Do they need to add more variables accordingly?    N.B. Ask participants to provide definitions for variables if needed. Ensure neutral wording of the variables.  Variables will be compared among each other to ensure **clarity of their meanings** and avoid duplication. |
| 60’ | Give an example on causal linkage of variables (IBO) – 2’  Causal loop diagram development  The facilitator (IBO) will write down the different states by which the population goes from being healthy to getting NCDs and then to control, complications or death.  Participants will be invited to add the variables to this model.  Prompt providers to tell us about their place and experience in this model.  What makes their job easy/hard? What problems do they see?  The research team will ensure that the causal links, proposed by the groups, are clear and justifiable by them.  Peer-review of the models by other group members will be encouraged. |
| 20’  30’ | Model presentation and Identification of points of fragility  Each group will present the model and the other groups will be invited to comment on the model.  The research team will ask the participants to identify points of ‘fragility’ in the system and to vote on their severity: 5 votes can be split however they want. |
| 45’ | Lunch |
| 60’ | Identification / prioritization of interventions  We want to know participants’ opinions on where and how to intervene in the models they have just created.   1. What are the elements of a good strategy for intervening? 2. Look at the points of fragility. How would you intervene? 3. Look at the criteria you elicited and at the interventions proposed. Can you classify them?   High Impact    Ease of implementation  (-) (+)  Low Impact  Giving out stipends for the day  Final payments |
